# Timing of Discharge Follow-up for Acute Pulmonary Embolism: Retrospective Cohort Study

**DOI:** 10.5811/westjem.2014.12.23310

**Published:** 2015-01-12

**Authors:** David R. Vinson, Dustin W. Ballard, Jie Huang, Adina S. Rauchwerger, Mary E. Reed, Dustin G. Mark

**Affiliations:** * The Permanente Medical Group, Oakland, California; † Kaiser Permanente Roseville Medical Center, Roseville, California; ‡ Kaiser Permanente Division of Research, Oakland, California; § Kaiser Permanente San Rafael Medical Center, San Rafael, California; ¶ Kaiser Permanente Oakland Medical Center, Oakland, California

## Abstract

**Introduction:**

Historically, emergency department (ED) patients with pulmonary embolism (PE) have been admitted for several days of inpatient care. Growing evidence suggests that selected ED patients with PE can be safely discharged home after a short length of stay. However, the optimal timing of follow up is unknown. We hypothesized that higher-risk patients with short length of stay (<24 hours from ED registration) would more commonly receive expedited follow up (≤3 days).

**Methods:**

This retrospective cohort study included adults treated for acute PE in six community EDs. We ascertained the PE Severity Index risk class (for 30-day mortality), facility length of stay, the first follow-up clinician encounter, unscheduled return ED visits ≤3 days, 5-day PE-related readmissions, and 30-day all-cause mortality. Stratifying by risk class, we used multivariable analysis to examine age- and sex-adjusted associations between length of stay and expedited follow up.

**Results:**

The mean age of our 175 patients was 63.2 (±16.8) years. Overall, 93.1% (n=163) of our cohort received follow up within one week of discharge. Fifty-six patients (32.0%) were sent home within 24 hours and 100 (57.1%) received expedited follow up, often by telephone (67/100). The short and longer length-of-stay groups were comparable in age and sex, but differed in rates of low-risk status (63% vs 37%; p<0.01) and expedited follow up (70% vs 51%; p=0.03). After adjustment, we found that short length of stay was independently associated with expedited follow up in higher-risk patients (adjusted odds ratio [aOR] 3.5; 95% CI [1.0–11.8]; p=0.04), but not in low-risk patients (aOR 2.2; 95% CI [0.8–5.7]; p=0.11). Adverse outcomes were uncommon (<2%) and were not significantly different between the two length-of-stay groups.

**Conclusion:**

Higher-risk patients with acute PE and short length of stay more commonly received expedited follow up in our community setting than other groups of patients. These practice patterns are associated with low rates of 30-day adverse events.

## INTRODUCTION

Historically, emergency department (ED) patients with acute pulmonary embolism (PE) in the United States have been admitted for at least several days of inpatient care.^1^,^2^ In addition to the initiation of treatment, a multi-day inpatient stay allows for the prompt detection of disease extension and treatment complications. It also provides opportunities for important patient education prior to discharge.

Growing evidence suggests that carefully selected low-risk patients with PE can be safely discharged home either directly from the ED or after a short hospital stay (<24 hours).^3^–^5^ Truncating the typical inpatient stay limits the time available for patient observation and education. Timely post-discharge follow up, however, may serve to mitigate this loss by providing opportunities for urgent patient re-evaluation and education reinforcement. In fact, outpatient management of patients with acute PE is inadvisable without both “well-developed patient education” and “adequate support and follow-up mechanisms for the discharged patient,”^6^,^7^ key elements of a safe transition of care.^8^–^10^ However, the optimal nature and timing of follow up after expedited discharge for these patients has not been established.

Prospective studies of the outpatient management of PE vary widely in their timing and frequency of scheduled follow up. The reports range, on the one hand, from daily phone contact for seven consecutive days following discharge^11^ to no pre-arranged contact with a clinician until an outpatient appointment one week after discharge.^12^ Retrospective descriptive studies of outpatient management programs for ED patients with PE also vary considerably in their follow-up strategies.^13^,^14^ Follow-up practice patterns from ‘real life’ community settings have not been reported.

We hypothesized that higher-risk outpatients with acute PE being discharged home after a short hospital length of stay (<24 hours from ED registration) would more commonly receive expedited follow up (≤3 days) than their lower-risk or longer-stay counterparts. We undertook this study to describe the practice patterns of PE management in community hospitals and to evaluate the influence of length of stay, risk class, and site-of-discharge on the timing of post-discharge outpatient follow up and short-term outcomes.

## METHODS

### Study Design and Setting

This retrospective cohort study included adult ED patients who were diagnosed with acute PE between January 1, 2013 and May 31, 2013 in six community EDs within Kaiser Permanente (KP) Northern California, a large integrated healthcare delivery system that provides comprehensive care for more than 3.4 million members. KP health plan members represent approximately 25–30% of the population in areas served and are similar to the general population with respect to race/ethnicity, socioeconomic status, and education.^15^,^16^ The study was approved by the KP Northern California Health Services Institutional Review Board.

The EDs had an annual census in 2013 from 26,000 to 85,000 and were staffed by residency-trained, board-certified emergency physicians. The medical centers had inpatient bed capacities ranging from 50 to 325. Inpatient care is provided by board-certified internists, all of whom are hospitalists. Three medical centers were satellite sites for residency training programs and had residents rotate to various degrees through their emergency and hospitalist departments.

During 2013, all facilities had 24/7 access to on-site computed tomography pulmonary angiography with around-the-clock interpretation by board-certified radiologists. Formal compression ultrasonography and ventilation perfusion imaging were not available during late night hours. Two facilities had a designated clinical decision area, functioning akin to a short-stay (<24 hours) observation unit, managed by hospitalists. Initial site-of-care decisions and total length of stay were in the hands of the treating physicians; no clinical care pathways for PE were in effect.

All facilities provided pre-discharge patient education regarding the disease and its treatment and had pharmacy available around-the-clock for discharge medications and supplemental patient education. Treating physicians commonly employed the standard KP Northern California discharge orderset for thromboembolism, which recommends warfarin with concomitant bridging therapy using enoxaparin. Alternative oral anticoagulants approved for the treatment of PE were not often prescribed, as the formulary restricts their use to patients who have failed or are unable to adhere to warfarin. Outpatient warfarin dosing was managed by each facility’s pharmacy-led anticoagulation service. The percent time in therapeutic international normalized ratio range at these facilities in 2013 was a respectable 72% to 74% (the higher the percentage, the higher the quality of care and the better the clinical outcomes).^17^–^19^

Throughout the study period no follow-up policy was in effect at any of the medical centers for patients with acute PE who were discharged home. The timing and nature (telephone vs clinic) of the follow-up appointment with the patient’s primary care provider was arranged at the discretion of the discharging clinician, who either directly provided the follow-up appointment or recommended the patient arrange it themselves within a certain time frame. These patient-driven access appointments were secured either via a 24/7 telephone appointment call center, an email directly to the patient’s primary provider, or by electronically booked appointment times available through the patient portal of kp.org.^20^–^22^

### Selection of Participants

Non-gravid ED patients aged 18 years or older were included if they had an acute PE that was objectively confirmed by radiographic imaging, performed either in the ED or within the 12 hours prior to ED arrival, and no recent diagnosis of acute venous thromboembolism in the prior 30 days. Objective diagnostic confirmation was based on the final interpretation by a board-certified radiologist (or nuclear medicine physician, as indicated). We also included patients with a compression ultrasound positive for deep vein thrombosis in conjunction with respiratory complaints consistent with acute PE, as other outpatient PE research studies have done.^11^,^23^,^24^

Patients with the following conditions were excluded from further analysis because follow up within the integrated care system was not possible or customary: discharge to a skilled nursing facility, non-members, as they received follow-up care outside our delivery system, and those who died in the ED or during hospitalization.

### Data Collection

Investigators used a standard computerized data collection tool that combined extracted administrative data with manual chart review of the comprehensive integrated electronic health record.^25^ Patient-level clinical data was electronically accessible within hierarchical databases as described previously.^26^ We assessed risk for all-cause 30-day mortality using the PE Severity Index, the most well-studied validated risk stratification tool available.^11^,^27^ We chose this prognostic tool because it is recommended by international society guidelines as a safe and effective means of identifying patients eligible for outpatient management.^28^,^29^ We calculated the ED PE Severity Index score using the worst, and not the first, ED vital signs. We also included qualifying pre-arrival vitals from the clinic or emergency medical services that were documented in the physician notes. Patients with scores ≤85 points were classified as low risk (<5%) for 30-day mortality and those with scores above 85 as higher risk (>5%), based on published data.^11^,^27^

An outpatient appointment qualified as a clinician follow up if the provider (physician or nurse practitioner) who evaluated the patient was a generalist or a specialist in pulmonary medicine or hematology/oncology. Timing was measured in days since discharge and included both in-person and telephone visits. We excluded Internet-based secure messages between patients and their providers.^20^,^22^,^30^

### Outcome Measures

Our primary outcome measure was an expedited post-discharge follow up ≤3 days of discharge. A three-day endpoint defined expedited follow up since it represents the conservative end of the range in the outpatient PE literature^3^ and is commonly used in research on telephone follow up, both after hospitalization and ED care.^31^,^32^

Unscheduled return ED visits ≤3 days included ED visits for any reason that were not initially arranged at the index visit. Five-day readmissions were counted as PE-related if any of the following were noted: complaints of dyspnea, chest pain, syncope, leg pain, or bleeding; findings of pleural effusion, elevated liver enzymes, new anemia or hemorrhage, new or worsening deep vein thrombosis or PE; or one of the following interventions: respiratory support (non-rebreather mask, non-invasive ventilation, endotracheal intubation, or mechanical ventilation), parenteral vasopressors, inferior vena cava filter placement or removal, or cardiopulmonary resuscitation.

Multiple processes were instituted to enhance the accuracy and reliability of the data abstraction process. All abstractors received training on the content and coding of each data element, data handling and data transmission procedures, as well as protocols to respond to questions or problems during the study. The principal investigator (DRV) monitored day-to-day data collection activities and answered coding questions. All complications were reviewed by two investigators for confirmation. Ambiguities in classification or diagnosis were arbitrated by a third investigator. Additionally, 15% of cases were randomly selected for independent review by a second investigator to assess for inter-rater reliability, reported as percent agreement, on the following variables: PE Severity Index score, risk class, site of discharge, expedited follow up, nature of the follow up, 3-day, 5-day, and 30-day outcomes.

### Statistical Analysis

Continuous variables are presented as means with standard deviation and categorical data are presented as the percentage of frequency of occurrence, with 95% CIs. We performed bivariate analysis to compare patients with expedited follow up (≤3 days) and those with non-expedited follow up (>3 days). P-values are shown for t-test or chi-squared test. A two-tailed p-value of less than 0.05 indicated statistical significance. In analyses stratified by PE Severity Index risk status, adjusted odds ratios (aORs) were calculated using multivariate logistic regression to determine whether length of stay <24 hours was associated with expedited follow-up after adjusting for age and sex. Tested covariates included age, sex, discharge from the ED or clinical decision area, and length of stay <24 hours from ED registration. Pairwise correlation for covariates was tested with a threshold r-value of less than 0.7 for inclusion in the model. The variance inflation factor was also determined for all variables in the regression model with an upper threshold of 10 for inclusion. We performed analyses using STATA v13.1 (StataCorp LP, College Station, Texas).

## RESULTS

We identified 203 cases of PE, 28 of which were excluded because of hospital discharge to a skilled nursing facility (n=15), non-member status (n=8), and inpatient death (n=5). The mean age of the remaining 175 patients was 63.2 (±16.8) years, and 87 (49.7%) were female. Overall, 56 patients (32.0%) were discharged within 24 hours.

The short and longer length-of-stay groups were comparable in age, sex, and rate of timely engagement with anticoagulation services, but differed significantly in their PE Severity Index risk classification and their site of discharge (Table 1). Overall, 93.1% (n=163) of our cohort received follow up within one week of discharge. One hundred patients (57.1%) received expedited follow up (≤3 days), most often by telephone (67/100).

We report the timing of initial post-discharge follow up stratified by risk class and length of stay in Table 2. This bivariate analysis suggests that higher-risk PE patients with short length of stay more commonly experienced expedited follow up compared with higher-risk patients with longer length of stay or low-risk patients with either short or longer length of stay.

Given the interaction between length of stay and risk class, we stratified the cohort by PE Severity Index class (higher risk vs. low risk) for the multivariate analysis. The association we found in bivariate analysis held up after controlling for age and sex. We found that short length of stay was independently associated with expedited follow up in the higher-risk patients (aOR of 3.5 [95% CI [1.0–11.8]; p=0.04]), but not the low-risk patients (aOR of 2.2, 95% CI [0.8–5.7]; p=0.11). Site of discharge (ED or clinical decision area) was collinear with short length of stay (r=0.8) and accordingly was not included in the regression models.

Of the 43 patients sent home from the ED or clinical decision area after a short stay, 23 were discharged from the ED and 20 from the clinical decision area. The rates of expedited follow up between these two sites-of-discharge groups were not statistically significant: ED (21/23) and clinical decision area (15/20).

Unscheduled return ED visits ≤3 days were uncommon (1.1%; 95% CI [0.1%-4.3%]), as were PE-related readmissions to the hospital ≤5 days (1.1%; 95% CI [0.1%-4.3%]), neither of which were significantly different between the short-stay and the longer-stay groups (Table 1). Rates of post-discharge all-cause 30-day mortality were also low (1.1%; 95% CI [0.1%-4.3%]) and not significantly different between the two groups.

The two patients who died were both Class V on the PE Severity Index, and hence at higher risk for 30-day all-cause mortality.^27^ One patient was a 52-year-old woman with advanced metastatic breast cancer who at hospital discharge was enrolled in hospice care. She died at home 12 days later. The other was an 87-year-old man with significant comorbidities whose index hospital course was complicated by a major lower gastrointestinal hemorrhage on day seven, requiring a transfusion of two units of red blood cells. He had an out-of-hospital asystolic arrest on day 30.

The inter-rater reliability results for the following eight variables ranged between 96% to 100% agreement: PE Severity Index score, risk class, site of discharge, expedited follow up, nature of follow up, 3-day, 5-day, and 30-day outcomes.

## DISCUSSION

This retrospective cohort study found that higher-risk patients with acute PE sent home within 24 hours of ED registration more commonly received expedited follow up within three days than low-risk patients and those of any risk category with longer lengths of stay. Given the relative novelty in the U.S. of home management of ED patients with acute PE, we suspected that physicians might feel the need to be more vigilant when sending higher risk patients home who had received only a short period of medical observation. Our results support this hypothesis.

The optimal timing of follow-up appointments for patients with acute PE who are discharged home is unknown, though this transition of care can be critical to patient safety, especially in the elderly.^8^–^10^ How a patient’s risk classification, site of discharge, or their comorbidities and psychosocial factors should influence the timing of follow up is also unknown. The timing of post-discharge follow up reported for this population varies. Prospective studies of outpatient PE management ensure telephone follow up as soon as the next day^11^ or wait for a week before seeing the patient in the clinic.^11^ Other prospective studies fall between these extremes.^3^ One established outpatient treatment protocol for ED patients with acute PE in Canada has their discharged patients seen in the thrombosis clinic in 24–48 hours.^13^ No published outpatient PE policy defers the initial follow up beyond the first week.

The nature of the initial post-discharge follow up also varies: some see their patients in person and others contact them by phone.^3^,^13^ If an element of the physical examination is crucial to the follow-up assessment, which is uncommon with PE, then an in-person clinic visit is preferred. Otherwise, a telephone conversation may be just as effective, despite the loss of face-to-face communication.^31^,^32^ A phone encounter offers greater patient convenience by reducing their outlay of time, travel, and costs. Telephone follow up has been demonstrated to improve patient satisfaction, as well, though its impact on clinical outcomes remains inconclusive.^33^,^34^ Video visits may offer a promising alternative, maintaining the convenience of a telephone visit with the advantages of virtual face-to-face communication.^21^,^35^

Follow-up appointments, either in person or over the telephone, allow for continuing patient education, encouragement of treatment compliance, management of symptoms, and the answering of questions. It is difficult to unravel the contribution made by timely follow up to the favorable outcomes associated with outpatient management of select patients with acute PE. Studies of home management all include careful post-discharge follow up one or more times within the first week as well as frequent telephone contact with anticoagulation services.^3^–^5^,^13^ We do not know if such low rates of adverse outcomes could have been achieved apart from timely patient reassessment during that first week after discharge. This is an important area for future investigation.

## LIMITATIONS

This study is subject to the limitations inherent in its retrospective design. Some of those shortcomings, however, are mitigated by our comprehensive electronic health record, our excellent capture of outcomes among KP health plan members, who seek care almost exclusively within the health plan, and the study’s high inter-rater reliability. Our limited sample size means the rates of adverse outcomes we measured are estimates with wide confidence intervals. The majority of our patients were discharged on warfarin and therefore also received close and careful monitoring by the pharmacy-led anticoagulation service. It is uncertain how clinic-based follow-up arrangements will change (or should change) for patients taking newer oral anticoagulants that don’t require efficacy monitoring.^36^

Our results may not be readily generalizable, as they reflect the practices of physicians who work within an integrated healthcare delivery system, where prompt outpatient follow up can be reliably arranged^37^ and our anticoagulation services carefully manage their patients. This tightly coordinated continuity of care may allow for shorter length of stay in the ED and inpatient units than in healthcare systems lacking reliable outpatient monitoring and follow up. Lastly, outpatient PE management is not altogether new in our healthcare system, having been practiced to a small degree for over a decade.^38^,^39^ Though more commonly employed in Europe and Canada, outpatient PE management has been uncommon in the U.S. A recent large PE registry from 22 EDs in the U.S. found that only 21 of 1,880 (1.1%) patients were discharged home from the ED without hospitalization.^2^

## CONCLUSION

In sum, we found that outpatients with acute PE nearly always received post-discharge follow up within the first week and over half received expedited follow up within three days. Higher-risk patients who were sent home within 24 hours of ED registration were more likely to receive expedited follow up. For all patients, the rate of adverse outcomes at both five days and 30 days was very low, though our study was not adequately powered to ensure the safety of this management approach. Short length of stay combined with expedited post-discharge follow up, however, appears to be a safe practice for selected patients in this integrated healthcare system. The effects of expedited follow up on patient satisfaction and clinical outcomes warrant further investigation.

## Figures and Tables

**Table 1:** Patient characteristics, management, and outcomes of patients with acute pulmonary embolism stratified by facility length of stay.

	Visit length of stay (n=175)		
	
	Short-stay (<24 hrs) n=56	Longer-stay (≥24 hrs) n=119	p-value
Patient characteristics	n (%)	n (%)	
Age years*	60.4 (18.4)	64.3 (16.1)	0.16
Sex female	24 (42.8)	63 (52.9)	0.28
Pulmonary Embolism Severity Index: low risk†	35 (62.5)	44 (37.0)	<0.01
Management			
Site of discharge			
Emergency department (ED) or clinical decision area	43 (76.8)	2 (1.7)	<0.001
Inpatient unit	13 (23.2)	117 (98.3)	
Expedited follow up with clinician (≤3d)	39 (69.6)	61 (51.3)	0.03
By telephone	25	42	0.78
In clinic	14	19	
Follow up with clinician ≤7d	53 (94.6)	110 (92.4)	0.83
Anticoagulation services			
Discharged on warfarin	53 (94.6)	107 (89.9)	0.45
Anticoagulation telephone contact ≤3d	50 (94.3)	99 (92.5)	0.92
Adverse outcomes			
Unscheduled ED visit ≤3d	1 (1.8)	1 (0.8)	0.58
Thromboembolism-related readmission to hospital ≤5d	1 (1.8)	1 (0.8)	0.58
Post-discharge all-cause mortality <30d	0	2 (1.7)	0.83
Sum of adverse events	2 (3.6)	4 (3.4)	

* Mean (SD).

† Low risk: Pulmonary Embolism Severity Index Class I or II (points ≤85); higher risk: Class III through V (points >85).

**Table 2:** Timing of initial post-discharge follow up stratified by risk class and length of stay for emergency department patients with acute pulmonary embolism (unadjusted).

	Timing of initial post-discharge outpatient follow up			
	
	Cases n	≤3 days n (%)	>3 days n (%)	p-value
Low-risk*				
Short-stay†	34	22 (65)	12 (35)	0.29
Longer-stay	44	22 (50)	22 (50)	
Higher-risk				
Short-stay	21	17 (81)	4 (19)	0.04
Longer-stay	74	39 (53)	35 (47)	

* Low risk: Pulmonary Embolism Severity Index Class I or II (points ≤85); higher risk: Class III through V (points >85).

† Short-stay: Time from emergency department registration to departure from the facility <24 hours; longer-stay: ≥24 hours.
